# Chemotherapy-Induced Oesophageal Stricture in a Child with Osteosarcoma: A Case Report

**DOI:** 10.1155/2010/240763

**Published:** 2010-06-27

**Authors:** Daichi Ishimaru, Takatoshi Ohno, Masato Maeda, Yutaka Nishimoto, Katsuji Shimizu

**Affiliations:** ^1^Department of Orthopaedic Surgery, Takayama Red cross Hospital, Tenman-cho 3-11, Takayama, Gifu 506-8550, Japan; ^2^Department of Orthopaedic Surgery, Gifu University School of Medicine, 1-1 Yanagido, Gifu 501-1194, Japan

## Abstract

Treatment with a combination of chemotherapy and radiotherapy is known to be associated with oesophageal stricture in both children and adults with malignancies. However, oesophageal stricture resulting from chemotherapy alone is a rare complication, with few reports on it. We experienced a rare paediatric case of oesophageal stricture caused by chemotherapy for osteosarcoma of the left distal femur. After completion of the chemotherapy course, the patient showed dysphagia caused by the oesophageal stricture and underwent balloon dilatation for the oesophageal stricture. After balloon dilatation, he was able to ingest solid foods, and the oesophagus was normal without any strictures at the last follow-up (20 months after ballooning). Therefore, oesophageal stricture should be considered as a complication of treatment with chemotherapy alone in children with malignancies.

## 1. Introduction

The cure rate of paediatric malignancies has improved with the advances in both chemotherapy and radiotherapy over the last 3decades, and the long-term survival rate is currently 80% [1]. Because the number of surviving children with malignancies is increasing, the complications involved in the treatment of paediatric malignancies are becoming clear [2, 3]. Oesophageal stricture is one of the reported complications [4, 5]. Oesophageal stricture in children treated for malignancies can occur because of the administration of antineoplastic agents, radiotherapy, candidiasis, graft versus host disease, and so forth [6]. In previous reports on oesophageal complications in children with malignancies, it was suggested that treatment with a combination of chemotherapy and radiotherapy probably results in oesophageal stricture. However, to the best of our knowledge, only few studies have reported that chemotherapy alone induced oesophageal strictures in children. Here, we report a rare case of oesophageal stricture in a child who was undergoing only chemotherapy for osteosarcoma.

## 2. Case report

**Figure 1:**
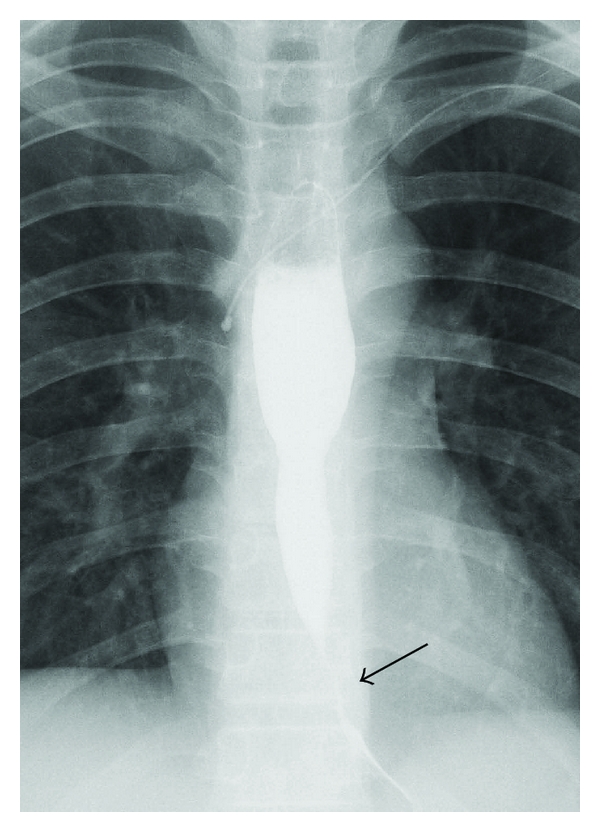
Chest radiograph obtained in the barium-swallowing examination showing stricture of the distal oesophagus. The black arrow indicates the oesophageal stenosis.

**Figure 2:**
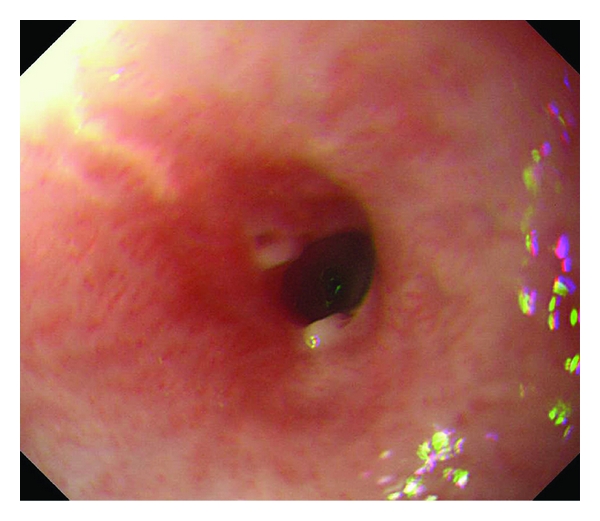
Upper gastrointestinal endoscopic image showing an annular stricture and slight reddish mucosa in the distal oesophagus.

A 14-year-old boy was diagnosed with osteosarcoma of the left distal femur. He underwent 4 courses of chemotherapy with cisplatin (CDDP) (167 mg) and doxorubicin (42 mg). He was also administered a 5-HT3 receptor antagonist to prevent nausea and vomiting and a regimen of the histamine-2-receptor antagonist to prevent gastroesophageal reflux disease; despite this, he complained of severe nausea and vomiting. The patient experienced hearing impairment because of CDDP, therefore, after the first 4 courses of chemotherapy, the regimen was changed to comprise ifosfamide (4200 mg), etoposide (84 mg), and methotrexate (28 mg). Two courses of this new regimen were administered. Five months after the diagnosis, the patient underwent extensive tumour resection and total knee replacement with a custom-made extendable knee replacement system (Growing KotzProsthesis;Stryker, Germany).Pathological examination showed that the tumour tissue was almost necrotic, which suggested that the preoperative chemotherapy was effective. After the operation, the patient was administered 6 courses of the second chemotherapy regimen over a period of 5 months. During this treatment period, he experienced nausea and vomiting. Four weeks after the postoperative 6-course chemotherapy, he complained of dysphagia and could not eat solid foods. A barium-swallowing examination showed significant narrowing of the distal oesophagus (Figure 1). Upper gastrointestinal endoscopy revealed stricture of the distal oesophagus, demonstrating slightly reddish and inflammatory mucosa at the distal oesophagus (Figure 2). The distal oesophagus was narrow, and the entire oesophageal wall was thick, so the endoscope could not pass beyond the stricture of the oesophagus. A biopsy of the strictured part of the lesion was not performed. He was diagnosed with chemotherapy-related oesophageal stricture, and balloon dilatation for the oesophageal stricture was performed 10 times over a period of 2 months. Finally, the oesophagus was dilated till its diameter increased by 12 mm. During this period, omeprazole (proton pump inhibitor, PPI) was administered. After ballooning, his dysphagia improved, and he could ingest solid food. At the final follow-up, which was conducted 20 months after the ballooning, the oesophagus was found to be normal with no strictures, and osteosarcoma of the left distal femur had also not relapsed and metastasized.

## 3. Discussion

Children with malignancies are likely to be exposed to many risk factors for oesophageal stricture during treatment, such as the administration of antineoplastic agents, radiation therapy, candidiasis, and graft versus host disease [2–8]. In particular, it is well known that radiation therapy is associated with the risk of oesophageal stricture in both children and adults [3]. Radiation injury to the oesophagus results in severe fibrosis, mucosal atrophy, and ischemic ulcerations and finally results in oesophageal stricture. Oesophageal stricture has also been reported to be caused by antineoplastic agents such as vinblastine, doxorubicin, 5-fluorouracil, and methotrexate [5, 9]. Moreover, some antineoplastic agents act synergistically with radiotherapy and exacerbate the oesophagitis associated with radiotherapy [2, 5]. Therefore, it was reported that chemotherapy combined with radiotherapy had a definite risk of oesophageal complications [10, 11]. In addition, candidiasis and graft versus host disease after bone marrow transplantation have been reported to contribute to oesophageal stricture in children [7, 8, 12]. With regard to oesophageal stricture in children with malignancies, Ellenhorn et al. [4] and Lal et al. [5] reported that 13 paediatric patients developed oesophageal strictures after treatment for malignancies at the same institution; of these, only 1 patient underwent chemotherapy alone, while the others received a combination of chemotherapy and radiotherapy. Kassam and Mandel [6] also reported a case of oesophageal stricture in a child who received only chemotherapy for treatment of a malignancy. Oesophageal stricture resulting from chemotherapy alone in paediatric patients with malignancies is very rare. In the case of our patient, antineoplastic agents were administered for the treatment of osteosarcoma of the left femur without radiotherapy, and bone marrow transplantation was not performed. Candidiasis might contribute to stricture of the distal oesophagus, but the gastrointestinal endoscopy examination did not show findings indicative of oesophageal candidiasis, such as mucosal ulceration and white mucosal plaque. Consequently, in our case, the oesophageal stricture seems to be a result of chemotherapy. It was reported that a number of antineoplastic agents, including doxorubicin, methotrexate, and cytosine arabinoside, show severe cytotoxicity against rapidly proliferating cells of the oesophagus by arresting the cell cycle [4]. Finally, the combined oesophageal toxicity of antineoplastic agents and peptic oesophagitis caused by frequent vomiting might have predisposed our patient to the oesophageal stricture. 

With regard to the treatment for oesophageal strictures in children, surgery, including oesophageal division, oesophageal diversion, and endoscopic balloon dilatation, is performed [5, 13]. Balloon dilatation is efficient for correcting oesophageal stricture in children, even though it has a low risk of oesophageal perforation [13, 14]. In severe cases where the patient is resistant to balloon dilation, aggressive surgical management is required [5]. In the case of our patient, gastrointestinal endoscopy did not reveal oesophageal fistula or ulcer, so it was suggested that the risk of oesophageal perforation in balloon dilatation is low. Finally, we performed balloon dilatation to correct the oesophageal stricture. 

In summary, we experienced a rare paediatric case of oesophageal stricture associated with chemotherapy. Chemotherapy alone seldom causes oesophageal stricture in children, but additional conditions such as vomiting may cause oesophageal stricture. Physicians should bear in mind that oesophageal strictures are one of the complications of chemotherapy in children with malignancies who are treated with chemotherapy alone. In the case of patients who exhibit persistent vomiting despite treatment using antinauseant or antacid agents, mild sedation may be effective for preventing oesophageal complications.
